# Phenotyping CCL2 Containing Central Amygdala Neurons Controlling Alcohol Withdrawal-Induced Anxiety

**DOI:** 10.3389/fncel.2020.580583

**Published:** 2020-09-18

**Authors:** Kathryn M. Harper, Darin J. Knapp, Caroline A. Todd, Irina Balan, Laure Aurelian, Hugh E. Criswell, George R. Breese

**Affiliations:** ^1^Department of Psychiatry, The University of North Carolina at Chapel Hill School of Medicine, Chapel Hill, NC, United States; ^2^Bowles Center for Alcohol Studies, The University of North Carolina at Chapel Hill School of Medicine, Chapel Hill, NC, United States; ^3^Department of Pharmacology, University of Maryland School of Medicine, Baltimore, MD, United States; ^4^Stanford University School of Medicine, Stanford University, Stanford, CA, United States; ^5^Department of Pharmacology, The University of North Carolina at Chapel Hill School of Medicine, Chapel Hill, NC, United States

**Keywords:** CCL2, neurons, ethanol, CCR2, CRF, CeA, CX3CL1, anxiety

## Abstract

Chemokines such as chemokine (C-C motif) ligand 2 (CCL2) play a role in several behaviors, including anxiety-like behavior, but whether neurons are an important source of CCL2 for behavior and how neuronal CCL2 may work to affect behavior are still debated. When a herpes simplex virus (HSV) vector was used to knockdown CCL2 mRNA in neurons of the central nucleus of the amygdala (CeA) in rats experiencing multiple withdrawals from low dose ethanol, anxiety-like behavior appeared in the social interaction task. To examine this finding further Fractalkine (CX3CL1), a chemokine that is often found to have an opposing function to CCL2 was measured in these rats. Both alcohol withdrawal and CCL2 knockdown increased the levels of the anti-inflammatory protein CX3CL1. The combination of alcohol withdrawal and CCL2 knockdown decreased CX3CL1 and may alter pro-inflammatory/anti-inflammatory balance, and thus highlights the potential importance of CCL2 and CCL2/CX3CL1 balance in anxiety. To find a mechanism by which neuronal chemokines like CCL2 could affect behavior, retrograde tracing with fluorescent nanobeads was done in two brain regions associated with anxiety the bed nucleus of the stria terminalis (BNST) and the ventral periaqueductal gray (VPAG). These studies identified CeA projection neurons to these brain regions that contain CCL2. To demonstrate that CCL2 can be transported *via* axons to downstream brain regions, the axonal transport blocker, colchicine, was given and 24 h later, the accumulation of CCL2 in CeA neuronal cell bodies was found. Finally, CCL2 in CeA neurons was localized to the synapse using confocal microscopy with enhanced resolution following deconvolution and electron microscopy, which along with the other evidence suggests that CCL2 may be transported down axons in CeA neurons and released from nerve terminals perhaps into brain regions like the BNST and VPAG to affect behaviors such as anxiety. These results suggest that neurons are an important target for chemokine research related to behavior.

## Introduction

Alcohol withdrawal increases brain neuroimmune proteins such as chemokine C-C motif ligand 2 (CCL2; He and Crews, 2008; Ehrlich et al., 2012; Freeman et al., 2012; Vetreno et al., 2013; Whitman et al., 2013; Kane et al., 2014; Harper et al., 2015) in a brain-region-specific fashion (Knapp et al., 2016; Baxter-Potter et al., 2017). Given that chemokines like CCL2 are thought to act as neuromodulators (Adler et al., 2006; Gruol, 2016), it has been suggested that CCL2 and similar chemokines can alter alcohol withdrawal-related behaviors. CCL2 has been linked to important alcohol-related behaviors, drinking (Blednov et al., 2005; June et al., 2015; Valenta and Gonzales, 2016), and alcohol withdrawal-induced anxiety-like behavior (Knapp et al., 2011; Harper et al., 2017) making it an important candidate target for the treatment of alcohol use disorder. The balance between pro-inflammatory chemokines like CCL2 and anti-inflammatory chemokines like fractalkine (CX3CL1) seems to be important to behavioral output such as alcohol drinking with CCL2 promoting drinking behavior while CX3CL1 inhibits drinking behavior (Aurelian and Balan, 2019). However, the role of CCL2 extends beyond alcohol-related behaviors and is related to other types of anxiety (Wohleb et al., 2013; Sawada et al., 2014). Perhaps CCL2 plays a role in anxiety in general.

CCL2 is expressed in neurons, microglia, and astrocytes (Banisadr et al., 2005a; June et al., 2015; Harper et al., 2017), and most studies to date have looked at expression changes caused by alcohol in tissues containing all three cell types. With few exceptions (Gruol, 2016; Harper et al., 2017), most of the studies examined the effect of altered CCL2 expression on behavior in a non-cell type-specific manner. In the amygdala, changes in CCL2 were shown to occur as early as 5 h into alcohol withdrawal (Harper et al., 2017, 2018). The central nucleus of the amygdala (CeA) has been shown to have changes in both CCL2 mRNA (Freeman et al., 2012) and the number of neurons containing CCL2 during alcohol withdrawal (Harper et al., 2017). Additionally, the CeA plays an important role in alcohol withdrawal-induced anxiety-like behavior (Huang et al., 2010). Changes in CCL2 in the neurons of the CeA have been correlated with changes in alcohol withdrawal-induced anxiety-like behavior (Harper et al., 2017). These changes suggest that CCL2 expressed in CeA neurons might lead to the anxiety-like behavior associated with alcohol withdrawal.

Little is known about the types of neurons that contain CCL2 in the CeA or how levels and release of CCL2 are controlled in these neurons. In the cerebellum, CCR2 (the receptor for CCL2) is almost exclusively found on neurons that also contain CCL2 (van Gassen et al., 2005), so it has been suggested that CCL2 regulates its own levels as do some other chemokines (Lim et al., 2016). Another potential candidate for CCL2 regulation is corticotropin-releasing factor receptor 1 (CRFR1), which is upregulated in response to alcohol exposure together with its ligand corticotropin-releasing factor (CRF; Eisenhardt et al., 2015). CRF and its receptor have also been found to control the toll-like receptor 4 (TLR4) signal, which includes CCL2 (June et al., 2015; Aurelian and Balan, 2019). CRFR1 antagonists were shown to block changes in CCL2 in the cortex (Whitman et al., 2013). Additionally, CRF and a CRFR1 antagonist also control alcohol withdrawal-induced anxiety-like behavior (Huang et al., 2010)—perhaps through CRF control of the activated TLR4 signal which controls CCL2. Though it is still unknown whether CRFR1 containing neurons are CCL2 containing neurons and thus CRF can control CCL2 by binding to the neuron that contains CCL2.

The CeA contains both interneurons and projection neurons. The type of neuron containing CCL2 in the CeA is unknown. In the spinal cord, CCL2 is made in the cell body of some neurons and transported to the terminals for release (Van Steenwinckel et al., 2011), but such precise localization was never done for CCL2 within brain neurons. If the CCL2 containing neurons in the CeA are projection neurons, this mechanism would allow the release of CCL2 into downstream brain regions.

This article sought to demonstrate whether loss of CCL2 solely in CeA neurons would alter alcohol withdrawal-induced anxiety-like behavior and whether this effect is related to changes in anti-inflammatory chemokines. Additionally, in an attempt to find potential regulators of CCL2 levels, we extended the co-localization of CCL2 with neuronal receptors with which it could interact. Retrograde tracing was done to determine whether CCL2 is found in projection neurons and potential downstream brain regions of these neurons. Finally, CCL2 was localized subcellular in neurons to help identify the potential mechanism whereby CCL2 affects behavior.

## Materials and Methods

### Animals

All protocols were approved by the UNC-Chapel Hill Institutional Animal Use and Care Committee. Male Wistar rats weighing 180–200 g were purchased from Charles Rivers (Raleigh, NC, USA). One cohort of rats underwent surgery for bilateral cannulation to be used for the viral vector studies before exposure to the alcohol paradigm (*N* = 7–11). The second cohort of alcohol naïve rats (*N* = 7) was used for CCR2 and CRFR1 colocalization immunohistochemistry experiments. Two additional cohorts of two naïve rats each were used for the nanobead injection experiments and the colchicine study. Finally, two naïve rats were used for confocal microscopy with enhanced resolution experiments and two for the electron microscopy experiments.

### Surgery and Brain Injections

**Figure 1:**
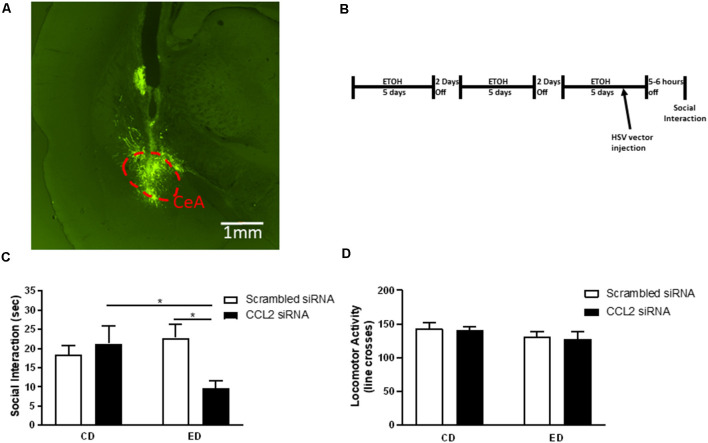
Loss of neuronal CCL2 during withdrawal from subthreshold alcohol causes anxiety-like behavior. **(A)** Representative injection site derived from post-mortem histological assessments of CeA injections. Neurons infected with herpes simplex virus (HSV) express GFP. The CeA is outlined by a red dashed line. **(B)** Diagram of injections/alcohol withdrawal paradigm. Male Wistar rats were subjected to three 5 day blocks of 4.5% w/v ethanol diet (ED) and were tested for social interaction (SI) 5–6 h into the third withdrawal. An amplicon with siRNA for CCL2 was injected into the CeA 2 days before the final withdrawal. **(C)** Only rats that received ethanol diet and loss of neuronal CCL2 showed reduced SI indicating increased anxiety-like behavior. **(D)** Neither virus nor ethanol diet alone or used in combination caused changes in locomotor activity. *N* = 7–11 per group. Data presented as mean ± SEM. *Post hoc* *p < 0.05.

Rats were anesthetized using isoflurane during stereotaxic surgery. The cannula and surgical screws (PlasticOne, Roanoke, VA, USA) were secured to the skull with acrylic cement. Coordinates used for CeA for cannula were AP −2.3, ML ±4.5, DV −5.5. The Herpes Simplex Virus (HSV) particles retain *in vivo* neurotropism (June et al., 2015). HSV-1 particles containing the amplicons pHSVsiMCP-1A and pHSVsiNCC were injected *via* the cannula. These amplicons were described previously (June et al., 2015) and are described in Supplementary Methods. 2.5 μl and 1.1 μl of pHSVsiMCP-1A and pHSVsiNCC respectively were injected over 10 min to get 2.5 × 10^5^ TUs into each CeA. It was noted previously that these amplicons do not have adverse effects (June et al., 2015). All brains were collected after testing to confirm injection location and for immunohistochemistry. Figure 1A shows an example of an injection site.

The second cohort of rats was also put under isoflurane during stereotaxic surgery. These rats received unilateral injections of 0.2 μl FluoSpheres™ (1:5 dilution with artificial cerebrospinal fluid) into either the bed nucleus of the stria terminalis (BNST; coordinates: AP −0.1, ML 1.6, and DV −6.6) or the ventral periaqueductal gray (VPAG; coordinates: AP −6.9, ML 3.05, and DV −6.4 angled 23.8°). Rats were allowed approximately 4 weeks for the nanobeads to extend through the neurons before tissue collection. Examples of injections can be seen in Figures 5D,H.

**Figure 2:**
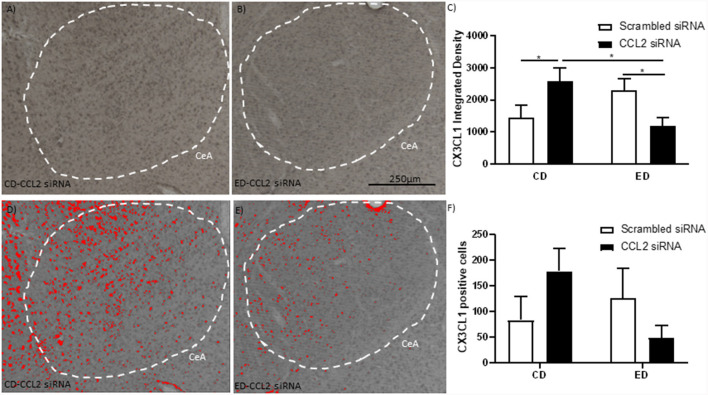
Alcohol withdrawal increases the anti-inflammatory CX3CL1 in the CeA, which is reversed by the loss of CCL2 in neurons. **(A)** Representative images are immunostained for CX3CL1 of the CeA of male Wistar rats that were microinjected with an amplicon with CCL2 siRNA. The CeA is outlined by a white dashed line. **(B)** Representative images are immunostained for CX3CL1 of the CeA of male Wistar rats that underwent alcohol withdrawal and microinjected with an amplicon with siRNA for CCL2. The CeA is outlined by a white dashed line. **(C)** The integrated density of CX3CL1 CeA images shows that alcohol withdrawal increases the levels of CX3CL1 which is reversed by the loss of CCL2. The images from panels **(A,B)** were converted into grayscale and then the same threshold was applied to both images to make the images in panels **(D,E,)**, respectively. The images in panels **(D,E)** highlight differences in identifiable CXC3CL1 containing neurons in the images using the same threshold. **(F)** Cell counts of CX3CL1 CeA images show that alcohol withdrawal increases the levels of CX3CL1 positive cells which is reversed by the loss of CCL2. *N* = 6 per group. Data presented as mean ± SEM. *Post hoc* *p < 0.05.

**Figure 3:**
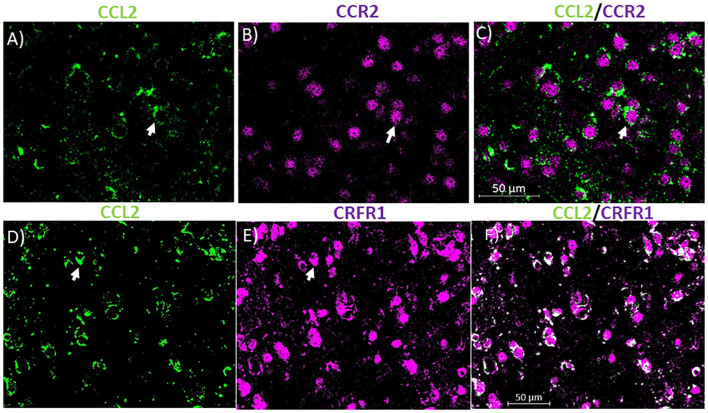
CCL2 containing neurons co-localize with CCR2 and corticotropin-releasing factor receptor 1 (CRFR1) in the CeA. **(A)** Representative images of the CeA neurons of naïve rats immunostained for CCL2. **(B)** Same image is immunostained for CCR2. **(C)** The merged image containing both immunostains showing neurons containing both CCL2 and CCR2 in the CeA. **(D)** Representative images of the CeA neurons of naïve rats immunostained for CCL2. **(E)** The same image is immunostained for CRFR1. **(F)** The merged image containing both immunostains showing neurons containing both CCL2 and CRFR1 in the CeA. White arrow indicates co-localization.

**Figure 4:**
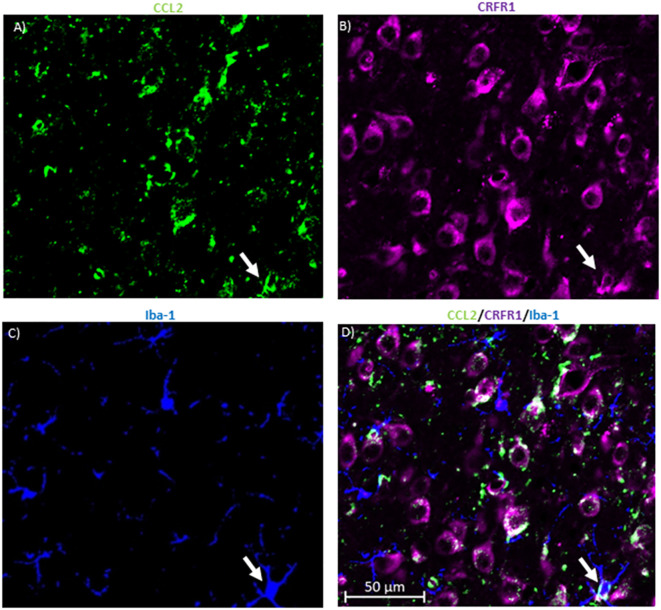
CRFR1 is not found in many microglia in the CeA. **(A)** Representative images of the cells in the CeA of naïve rats immunostained for CCL2. **(B)** The same image is immunostained for CRFR1. **(C)** Image of immunostaining of Iba-1 as a marker of microglia. **(D)** The merged image containing all markers shows microglia containing both CCL2 and CRFR1 in the CeA. White arrow indicates co-localization.

**Figure 5:**
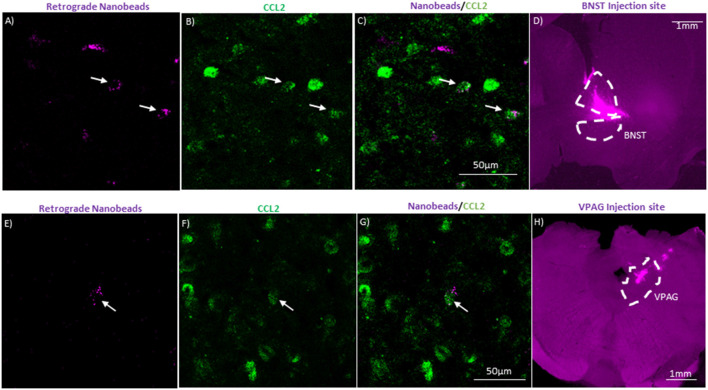
CeA projection neurons to the bed nucleus of the stria terminalis (BNST) and the ventral periaquaductal gray (VPAG) contain CCL2. **(A)** Representative image of CeA neurons in a naïve rat that underwent unilateral injection of fluorescent nanobeads into the BNST. **(B)** Same image immunostained for CCL2. **(C)** Merged image showing neurons containing both the fluorescent nanobeads and immunostained for CCL2. **(D)** Injection site of the fluorescent nanobeads in the BNST of the same rat. The BNST is outlined by a white dashed line. **(E)** Representative image of CeA neurons in a naïve rat that underwent unilateral injection of fluorescent nanobeads into the VPAG. **(F)** Same image immunostained for CCL2. **(G)** Merged image showing neurons containing both the fluorescent nanobeads and immunostained for CCL2. **(H)** Injection site of the fluorescent nanobeads in the VPAG of the same rat. The VPAG is outlined by a white dashed line. White arrow indicates colocalization.

The third cohort of rats was also put under isoflurane during stereotaxic surgery. These rats received unilateral cerebroventricular injections of 100 μg colchicine (MilliporeSigma, Burlington, MA, USA) in 10 μl of artificial cerebral spinal fluid or just artificial cerebral spinal fluid. Coordinates were AP −0.8, ML −1.6, and DV −4. After 24 h rats were perfused.

### Diets

Cannulated rats were first placed on a nutritionally complete control liquid diet (CD; Frye et al., 1983; Whitman et al., 2013; Harper et al., 2015). The following day, half the rats were placed on a 4.5% w/v ethanol diet (ED) that is isocaloric to the CD. Rats received three 5-day blocks of ethanol diet separated by 2 days of CD. The average alcohol consumed (g/kg/day) was 10.78 in the first block, 11.04 in the second block, and 9.6 in the third block. Previous work has shown that blood alcohol levels with 4.5% w/v ED range from 63 to 107 mg% (Wills et al., 2008). On the third day of the last 5-day block of ED, rats were given viral vector injections such that maximum expression of the siRNA was achieved by the day of social interaction testing (June et al., 2015). Five to six hours into withdrawal from the third 5-day block of ED, rats were tested on the social interaction test (Figure 1B). A two-way repeated-measures analysis of variance (RM ANOVA) showed there was no effect of virus type on alcohol consumed and no interaction between virus type and day of alcohol consumed (Supplementary Table S1). Rats showed weight gain across the ED paradigm and a three-way RM ANOVA showed there was no difference in weight gain between experimental groups (Supplementary Figure S1). This supports previous work using the 4.5%w/v ED (Overstreet et al., 2002).

### Behavioral Analysis

The social interaction test is an established measure of anxiety-like behavior. A reduction in social interaction reflects an elevated anxiety-like state (Overstreet et al., 2002; File and Seth, 2003; Knapp et al., 2011). Rats of approximately similar weight were paired, and the data from individual rats were used as prior reports demonstrated that the behavior across CD and ED groups is independent within pairs (Overstreet et al., 2002). Rats were paired for 5 min in a black plexiglass arena measuring 60 × 60 cm. All interactions were recorded so that an observer blind to treatment could score recordings. The time each rat engaged in social behavior (conspecific grooming, sniffing, following, crawling over/under its partner), and the number of lines crossed by two paws were recorded.

### Tissue Collection and Immunohistochemistry

Intraperitoneal injections of pentobarbital (100 mg/kg) were given before cardiac perfusion of 0.1 M cold PBS. Next, 4% paraformaldehyde in 0.1 M PBS was perfused. Tissue was sliced coronally at 40 μm using a VT 1000S vibratome (Lecia Biosystems, Buffalo Grove, IL, USA). Antigen retrieval was performed in sodium citrate buffer pH 6 for 30 min at 50°C. For CX3CL1 immunostaining 1:500 CX3CL1 (Life Technologies, Carlsbad, CA, USA) was used in combination with Vectastain anti-rabbit immunoassay kit (Vector, Burlingame, CA, USA). Four sections of the CeA between approximate Bregma −2.12 and −2.8 were captured with an Olympus BX51 (Olympus, Tokyo, Japan). Images were analyzed using Fiji ImageJ software (NIH, Bethesda, MD, USA). In the ImageJ program, images were converted to grayscale, and a threshold was determined. This threshold was applied to each image before measurements were taken within a standard 600 × 600 pixel square in the CeA (Wills et al., 2010).

Tissue for CCL2 colocalization experiments antigen retrieval was done at 50°C for 30 min in Sodium Citrate buffer pH 6 then tissue was blocked in 10% normal horse serum and stained for CCL2 1:100 (MilliporeSigma, Burlington, MA, USA). For CCR2 colocalization, CCR2 1:100 (Abcam, Cambridge, MA, USA) with the CCL2 antibody followed by goat anti-mouse Alexa flour 488 and goat anti-rabbit 633 secondaries at 1:500 (Thermo Fisher Scientific, Waltham, MA, USA). For CRFR1 colocalization, CRFR1 1:500 (Santa Cruz Biotechnology, Inc., Dallas, TX, USA) with the CCL2 antibody followed by rabbit anti-goat 488 and rabbit anti-mouse 594 secondaries at 1:500 (Thermo Fisher Scientific). For microglia colocalization, a microglia marker was used [Iba-1, 1:1,000 (Wako, Richmond, VA, USA)] with CRFR1 and CCL2 followed by chicken anti-rabbit 488, donkey anti-goat 594 (Thermo Fisher Scientific) and donkey anti-mouse 647 (Abcam, Cambridge, UK). For colocalization with nanobead, the CCL2 antibody was used followed by goat anti-mouse 488 (Thermo Fisher Scientific). Tissue from colchicine rats was stained for CCL2 1:200 (Santa Cruz Biotechnology, Inc., Dallas, TX, USA) and NeuN 1:500 (Millipore Sigma, Burlington, MA, USA). Secondary staining was done serially starting with rabbit anti-goat 594 1:1,000 followed by goat anti-mouse 488 1:500 (Thermo Fisher Scientific, Waltham, MA, USA). Tissue was placed in all primary antibodies overnight at 4°C on a rocker, and tissue was placed in all secondary antibodies for 1 h at 4°C on a rocker. All fluorescent images were captured with an LSM 780 Confocal Microscope (Carl Zeiss AG, Oberkochen, Germany). Microscopy was performed at the Neuroscience Center Microscopy Core Facility, supported, in part, by funding from the NIH-NINDS Neuroscience Center Support Grant P30 NS045892 and the NIH-NICHD Intellectual and Developmental Disabilities Research Center Support Grant U54 HD079124. Images were brightened using Fiji ImageJ software (NIH, Bethesda, MD, USA), colocalization was visualized by the fluorophore. Images for nanobead and CCL2 colocalization were stacked (Figures 5A–C,E–G). Gamma transformation was used on nanobead injection site images (Figures 5D,H) to assist in brightening up background tissue.

### Tissue Collection and Immunohistochemistry for Confocal Microscopy With Enhanced Resolution Following Deconvolution

Tissue was collected and sliced in the same manner as used in the immunohistochemistry for colocalization section described above. Tissue was blocked with 10% horse serum for 50 min. Sections were stained for CCL2 1:100 (Torey Pines Biolabs, Inc., Secaucus, NJ, USA) and PSD-95 1:500 (Novus Biologicals, Centennial, CO, USA) followed by chicken anti-rabbit 488 and chicken anti-mouse 594 secondaries at 1:500 (Thermo Fisher Scientific, Waltham, MA, USA). All images were captured with an FV3000RS Confocal Microscope (Olympus, Tokyo, Japan) equipped with a 60×/1.4 N.A. Plan ApoN oil lens and GaAsP detectors. After the acquisition, images were deconvoluted using Olympus FV31S-SW software with the ADVMLE (advanced maximum likelihood estimation) algorithm. Microscopy was performed at the UNC Neuroscience Microscopy Core Facility, supported, in part, by funding from the NIH-NINDS Neuroscience Center Support Grant P30 NS045892 and the NIH-NICHD Intellectual and Developmental Disabilities Research Center Support Grant U54 HD079124.

### Tissue Collection and Immunohistochemistry for Electron Microscopy

Rats received intraperitoneal injections of pentobarbital (100 mg/kg). 0.1 M cold PBS was perfused through the brain followed by 4% paraformaldehyde in 0.1 M PBS with 0.2% glutaraldehyde. Tissue was sliced coronally at 40 μm using a VT 1000S vibratome (Lecia Biosystems, Buffalo Grove, IL, USA). 0.3% H_2_O_2_ was used to block endogenous peroxidase activity. Tissue was blocked in 10% normal horse serum and stained for CCL2 1:500 (Torrey Pines Biolab Inc., Secaucus, NJ, USA) followed by a Vectastain anti-mouse immunoassay kit (Vector, Burlingame, CA, USA). Free-floating sections were then postfixed with 1% osmium tetroxide. Sections were embedded in Polybed 812 epoxy resin and ultrathin sections were cut on a Leica Ultracut UCT ultramicrotome (Leica Microsystems Inc., Buffalo Grove, IL, USA). Sections were collected on mesh copper grids and stained with Reynolds’ lead citrate (Reynolds, 1963). Images were acquired on a JEOL JEM-1230 transmission electron microscope (JEOL, Peabody, MA, USA) and digital images acquired using a Gaton Prius SC1000 CCD camera and Gatan Microscopy Suite 3.0 software (Gatan, Inc., Pleasanton, CA, USA). Imaging and sample preparation was done at the Microscopy Services Laboratory, Department of Pathology and Laboratory Medicine, which is supported in part by P30 CA016086 Cancer Center Core Support Grant to the UNC Lineberger Comprehensive Cancer Center.

### Statistical Analysis

Data were analyzed by two-way (virus and diet) ANOVA. Following a significant ANOVA, Fishers least significant difference (LSD) was used for individual *post hoc* comparisons. *P*-values <0.05 were considered significantly different. All data are displayed as mean ± SEM.

## Results

### CCL2 Containing Neurons in the CeA Are Involved in Alcohol Withdrawal-Induced Anxiety-Like Behavior

Given previous work suggesting that changes in neuronal CCL2 are correlated with anxiety-like behavior (Harper et al., 2017), a vector (amplicon) was used to selectively deliver CCL2 siRNA to neurons thereby knocking down CCL2 only in these cells (June et al., 2015). When the amplicon was given 3 days before the final withdrawal in the chronic intermittent alcohol (CIA) paradigm (Figure 1B), a significant interaction between virus and diet was observed (*F*_(1,33)_ = 5.71, *p* < 0.05; Figure 1C). Anxiety-like behavior increased in rats that received ED and the HSV vector for CCL2 siRNA compared with rats that received only ED or only the HSV vector for CCL2 siRNA (ED-CCL2 vs. CD-CCL2 *p* < 0.05; ED-CCL2 vs. ED-Sc *p* < 0.05). No significant effect of HSV vector type, diet, or interaction between HSV vector type and diet on locomotor activity was observed (Figure 1D).

### Alcohol Withdrawal and CCL2 Knockdown Alter the Levels of CX3CL1 in Neurons

CX3CL1 is an anti-inflammatory protein that has been linked to behavior and is associated with CCL2 (Aurelian and Balan, 2019). The levels of CX3CL1 in the CeA were measured in rats that underwent the behavioral testing discussed above to determine if shifts in the levels of CXC3CL1 in response to alcohol withdrawal and CCL2 knockdown could explain differences in behavior (Figures 2A–F). A significant interaction between alcohol withdrawal and CCL2 virus in the intensity of the CX3CL1 staining was observed (*F*_(1,20)_ = 9.5, *p* < 0.01; Figure 2C). CX3CL1 staining was higher in the rats that underwent alcohol withdrawal and in the rats that received the HSV vector for CCL2 siRNA whereas rats that were exposed to both alcohol withdrawal and received the HSV vector for CCL2 siRNA had the lowest levels of CX3CL1 staining (CD-Sc vs. CD-CCL2 *p* < 0.05; ED-CCL2 vs. ED-Sc *p* < 0.05). A similar nonsignificant trend was observed for the number of neurons positive for CX3CL (Figure 2F).

### Unlike Microglia Most CCL2 Containing Neurons in the CeA Contain CCR2 and CRFR1

To identify potential receptors in the CeA that control the levels of CCL2 in neurons, colocalization studies were done with CCL2. In the cerebellum, CCL2 colocalizes with CCR2 (van Gassen et al., 2005). It has been suggested therefore that CCL2 influences its own levels like some other chemokines (Lim et al., 2016). 72.12 ± 5.5% of CCL2 containing neurons in the CeA also contained CCR2 while 72.73 ± 3.5% of CCR2 containing neurons also contain CCL2 (Figures 3A–C). Because CRF had been shown to control the levels of CCL2 (Whitman et al., 2013) colocalization studies were also done with CCL2 and CRFR1. It was found that 78.96 ± 7.7% of CCL2 containing neurons in the CeA also contained CRFR1 while 63.96 ± 11.7% of CRFR1 containing neurons also contained CCL2 (Figures 3D–F).

To determine whether this colocalization is unique to neurons, the same colocalization studies were done with the microglial marker ionized calcium-binding adaptor molecule 1 (Iba-1; Figures 4A–D). The data indicate that 27.18 ± 5.39% of the microglia in the CeA contained CRFR1 while only 19.73 ± 5.72% of microglia in the CeA contained both CRFR1 and CCL2.

### CeA Projection Neurons Contain CCL2

Though it is known that CCL2 is found in CeA neurons (Banisadr et al., 2005a; Harper et al., 2017; June et al., 2015), it is unknown whether the CCL2 containing neurons are interneurons or projection neurons. Being localized in CeA projection neurons opens up the possibility that CCL2 made in CeA neurons could have effects on anxiety-like behavior by being released in other brain regions. To test whether CeA projection neurons contain CCL2, retrograde fluorescent nanobeads were injected into two brain regions associated with anxiety-like behavior the BNST (Gungor and Paré, 2016; Shackman and Fox, 2016) and the VPAG (Bertotto et al., 2010; Bonassoli et al., 2011, 2013). Nanobeads injected into the BNST were found in the CeA (Figures 5A–D). Additionally, retrograde fluorescent nanobeads injected into the BNST colocalized with CCL2 in the CeA. Retrograde fluorescent nanobeads injected into the VPAG were found in the CeA (Figures 5E–H). Additionally, retrograde fluorescent nanobeads injected into the VPAG colocalized with CCL2 in the CeA.

### CCL2 Is Transported Down the Axon

**Figure 6:**
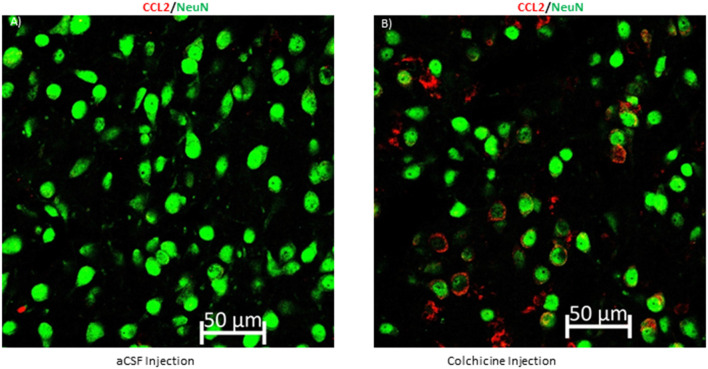
Blocking axonal transport increases CCL2 in neuronal cell bodies in the CeA. **(A)** Representative image of a rat that received an intracerebroventricular injection of artificial cerebral spinal fluid (aCSF) and **(B)** a rat that received colchicine, an axonal transport blocker.

As CCL2 has been localized to CeA projection neurons, the question remains if CCL2 can be transported down the axon of these projections neurons so that it can affect the BNST and the VPAG. This type of transport has been seen for CCL2 in the spinal cord but has yet to be confirmed in the brain (Van Steenwinckel et al., 2011). To confirm that CCL2 is transported down the axon of CeA neurons colchicine, an axonal transport blocker or artificial cerebral spinal fluid was given intracerebroventricularly to rats. CCL2 was found to accumulate in the cell body of CeA neurons 24 h after the colchicine was given as compared to rats that received injections of artificial cerebral spinal fluid (Figure 6).

### CCL2 Is Localized to Nerve Terminals in the CeA

**Figure 7:**
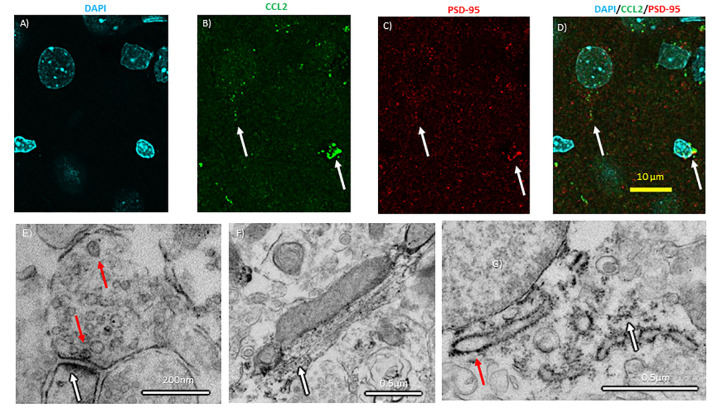
Subcellular localization of CCL2 within CeA neurons. **(A)** Neuronal nuclei in the CeA of naïve rats were stained with DAPI (4′,6-diamidino-2-phenylindole). **(B)** The same neurons were immunostained with CCL2. **(C)** Neurons were immunostained with PSD-95 (postsynaptic density 95) as a marker of synapses. **(D)** Combined images with all three markers. White arrows indicate colocalization. **(E)** Electron micrographs show CCL2 was found in vesicles (red arrow) in neuronal processes near postsynaptic densities (white arrow). **(F)** Additionally, DAB precipitate was found along microtubules (white arrow). **(G)** The rough endoplasmic reticulum (red arrow) and the surrounding ribosomes (white arrow) also show DAB precipitate.

In the spinal cord, CCL2 is transported down the axon allowing for vesicular release from the axon terminal (Van Steenwinckel et al., 2011). The colchicine experiments suggest that at least CCL2 axonal transport is possible in the brain, but CCL2 has yet to be localized to synapses in the brain. If true of the brain, CCL2 made in CeA neurons could be released into other brain regions. To test this hypothesis, confocal microscopy with enhanced resolution following deconvolution was used to colocalize CCL2 with postsynaptic density protein 95 (PSD-95), a marker for the post-synaptic density. CCL2 was found near PSD-95 (Figures 7A–D).

Electron microscopy was used to subcellularly localize CCL2 within the neuron in the CeA. CCL2 was localized to vesicles at the synapse in the CeA (Figure 7E). Additionally, CCL2 was found near microtubules and around the rough ER (Figures 7F,G).

## Discussion

When rats were tested for anxiety-like behavior in the social interaction test 2 days after microinjection of a siRNA to deplete CCL2 selectively in CeA neurons, there was no significant effect of the CCL2 depletion itself on social interaction. However, when the CCL2 depletion was paired with a mild withdrawal from chronic alcohol that by itself did not cause anxiety-like behavior, the combination caused a significant anxiety-like response. These results suggest the involvement of CCL2 in withdrawal-induced anxiety-like behavior, in agreement with previous work (Knapp et al., 2011). Interestingly, the previous study found that acute ICV injection of CCL2 increased withdrawal-induced anxiety-like behavior while the present study found that more chronic depletion of CCL2 in CeA neurons had the same effect. This difference in results between this study and previous work could be explained by the CCL2 in the neurons of the CeA somehow inhibiting anxiety-like behavior maybe by releasing CCL2 that binds to its receptors in the CeA or in downstream brain regions, but the effect of CCL2 binding to its receptors in other brain regions is to cause anxiety-like behavior. Another explanation would be that too much or too little CCL2 causes anxiety-like behavior. Withdrawal from low dose ethanol in this rat strain was not previously observed to cause increases in CCL2 levels in other brain regions (Harper et al., 2015), therefore the HSV vector will knockdown CCL2 levels below control conditions. This second explanation would also explain why knockdown of CCL2 mRNA in neurons of the CeA of P rats by the same HSV used in this article, blocked another alcohol-related behavior, alcohol self-administration (June et al., 2015). P rats have innately elevated levels of CCL2 (Eisenhardt et al., 2015), so knockdown of CCL2 in these rats would perhaps return CCL2 levels closer to other rat strains like the Wistar strain used in this article. If this is true one might speculate that if a higher concentration of ethanol is used in this paradigm, a concentration that is more likely to cause increases in CCL2 in the CeA (Harper et al., 2015) as well as a concentration of ethanol that can in and of itself cause anxiety-like behavior, that knockdown of neuronal CCL2 could reverse the anxiety-like behavior. It would additionally be interesting to see if other behaviors that are induced by stimuli that increase CCL2 and have also been shown to be affected by loss or antagonism of CCR2 such as stress-induced anxiety (Wohleb et al., 2013) and methamphetamine conditioned place preference (Wakida et al., 2014), would also be blocked by this HSV vector.

It’s important to note that this study, which was not designed to measure changes in alcohol drinking, found no effect of the CCL2 knockdown on alcohol consumption (Supplementary Table S1). However, alcohol consumption in the present case was not voluntary as the liquid ethanol diet was included in the animal’s food source. Another study did find that the HSV vector for CCL2 siRNA decreased voluntary alcohol drinking in P rats (June et al., 2015). That study used a specific strain of rat bred for high alcohol consumption and having innately elevated CCL2 levels.

The balance of pro-inflammatory CCL2 and anti-inflammatory CX3CL1 is thought to be important for the regulation of behaviors associated with alcohol drinking (Aurelian and Balan, 2019) and alcohol use disorder (García-Marchena et al., 2017). Both CCL2 and CX3CL1 are found in neurons in several brain areas, such as the amygdala (Tarozzo et al., 2003; Banisadr et al., 2005a). Therefore, the interaction between these two chemokines was examined in the present paradigm. The knockdown of CCL2 in the CeA increased the intensity of the CX3CL1 staining, but the number of CeA cells positive for CX3CL1 did not increase, indicative of increased neuronal CX3CL1 expression. This expression might be due to a TLR4 signal, which under normal conditions controls the levels of both CCL2 and CX3CL1 to create a balance between them (Aurelian and Balan, 2019). A similar trend was observed in rats that underwent alcohol withdrawal which is known to affect CCL2 most likely through a distinct TLR4 pathway (June et al., 2015; Aurelian and Balan, 2019). These alcohol-related effects on the TLR4 could also affect CX3CL1 expression. Indeed, it was found that the increase in CX3CL1 caused by CCL2 knockdown was completely reversed following withdrawal from alcohol suggesting that the loss of both CCL2 and CX3CL1 below functional levels may be responsible for the increase in anxiety-like behavior in this experimental group. According to this interpretation under normal conditions, CX3CL1 could balance CCL2 and function as an inhibitor of anxiety. Altogether this confirms the role of CCL2 in behavior and to clarify the effects of CCL2 on behavior, the possible mechanisms for its behavioral effect were explored in the last section of this article.

Given the importance of CCL2 levels in the CeA in anxiety-like behavior, it is important to identify the upstream pathways that regulate CCL2. CCL2 binds to its own receptor CCR2 (Huang et al., 2010; Harper et al., 2018). In agreement with previous findings in cerebellar Purkinje cells, most of the CCL2 containing neurons, in the CeA also expressed CCR2. However, it remains unclear whether CCL2 is released from the cell body, indicative of an autocrine/paracrine function, or CCL2 is released from nerve terminals of other CCL2 containing neurons in the CeA or neurons projecting to the CeA. In the case of Purkinje neurons and the CeA neurons in this article, CCR2 staining was most prominent in the perinuclear region, a finding suggesting the storage of nonfunctional receptors (van Gassen et al., 2005). A limitation of this study is that colocalization of CCL2 and CCR2 were only measured in naïve rats. To date changes in CCR2 in the CeA in response to stressors have been underexplored. Future studies should look at both the response of CCR2 to alcohol and stress and how this changes CCR2’s colocalization with CCL2.

Another, perhaps distinct pathway, that has been suggested to control CCL2 levels is CRF. A CRF antagonist has been shown to block alcohol-induced increases in CCL2 in the whole cortex (Whitman et al., 2013). Additionally, a CRF antagonist blocked alcohol-induced increases in TLR4 in the CeA, a finding that was suggested to reflect an upstream mediator of the increases in CCL2 in that model (June et al., 2015). Finally, CRF like CCL2 can induce anxiety-like behavior in combination with subthreshold alcohol withdrawal (Huang et al., 2010). Given that CRFR1 are found on neurons, microglia, and astrocytes (Kapcala and Dicke, 1992; Stevens et al., 2003), CRF could be binding directly to neurons to affect neuronal CCL2 levels, or CRF could be binding to one of the other cell types and acting through this cell type to indirectly affect neuronal CCL2 levels. The colocalization of the CRFR1 and CCL2 in neurons in the CeA suggests that CRFR1 might be controlling CCL2 levels in CeA neurons by binding to the CRFR1 on those CCL2 containing neurons. Once the CRF binds neuronal CRFR1, a pathway might be initiated that leads to increased CCL2 through TLR4 (June et al., 2015; Aurelian and Balan, 2019). A limitation of this study was that colocalization of CCL2 and CRFR1 was only measured in naïve rats. Future studies should look at changes in the colocalization of CCL2 and CRFR1, which might indicate a shift in the ability of CRF to control CCL2 levels in response to stressors.

CRFR1/Iba1 colocalization data do not exclude the possibility that CRF could be binding to the microglia as about 27% of identified microglia had CRFR1, but given that there is clearly more CRFR1 on neurons, this possibility would seem less likely. Additionally, only 20% of microglia, at least in the CeA, contain both CRFR1 and CCL2. The small number of microglia that contain CCL2 and CRFR1 makes it unlikely that the results with the antagonists involve a direct effect of CRF on CCL2 from a microglia source instead of a neuronal source. Even if CRFR1 antagonists are binding to microglia their effect on CCL2 would most likely be on neuronal CCL2 level. It is also unlikely that the source of CCL2 that is related to alcohol withdrawal-induced anxiety-like behavior are microglia as minocycline, an inhibitor of microglial activation, given both acutely during the final withdrawal and chronically during all three withdrawals was not able to reverse the effects of alcohol withdrawal on anxiety-like behavior (Harper et al., 2018). Minocycline did inhibit the alcohol withdrawal-induced increase of CCL2 mRNA in the amygdala when given in extended withdrawal (23 h into withdrawal), but not during acute withdrawal (5–6 h into withdrawal when anxiety-like behavior is at its peak; Harper et al., 2018). Altogether, these data might indicate that the microglia might be related to later changes in CCL2 such as a source of CCL2 for the negative feedback loop discussed above that shuts off CCL2 production in neurons by binding to neuronal CCR2 receptors instead of as a source of CCL2 involved in anxiety-like behavior.

The confocal microscopy with enhanced resolution following deconvolution and the electron microscopy data are the first to subcellularly localize CCL2 to synapses on CeA neurons, consistent with previous findings in the spinal cord (Van Steenwinckel et al., 2011). The electron microscopy results also localize CCL2 in the cytoplasm and in the microtubules, consistent with previously published CCL2 immunohistochemistry data (Banisadr et al., 2005a; June et al., 2015; Harper et al., 2017). The cytoplasmic staining most likely represents the production of CCL2 and the microtubule staining is the transport of CCL2 to the synapses at nerve terminals, which is once again consistent with what is found in the spinal cord (Van Steenwinckel et al., 2011). This conclusion is furthered supported by the colchicine studies. Colchicine blocks transport through microtubules and therefore blocks axonal transport, so after administrating colchicine proteins that would normally be moved down the axon accumulate in the cell body (Breder et al., 1993). Localizing CCL2 at synapses suggests that CCL2 produced by neurons in the CeA might be released in downstream brain regions such as the periaqueductal gray, where CCR2 receptors are found on neurons (Banisadr et al., 2005b). This is further supported by our finding that projection neuron from the CeA to regions related to anxiety, like the BNST and the VPAG, contain CCL2. In other brain regions, CCL2 has been shown to have electrophysiological effects (van Gassen et al., 2005; Zhou et al., 2011), therefore the CCL2 changes in neurons in the CeA caused by stimuli like alcohol (Harper et al., 2017) could lead to electrophysiological changes in downstream brain regions like the periaqueductal gray. Electrophysiological changes in this region could lead to behaviors that are associated with alcohol use. Altogether these actions may represent a neuronal CCL2 circuit that could be involved in behaviors such as anxiety. However, these results do not exclude the possibility that CeA neuronal CCL2 could be affecting behavior by electrophysiologically affecting CeA neurons, perhaps through CCL2 being released from CeA interneurons. Future investigations should further delineate the types of neurons that contain CCL2 and include electrophysiological studies with CCL2 to help to address these questions.

Collectively these results confirm the importance of neuronal CCL2 in behavior and highlight the need for further exploration of the CCL2/CCR2 neuronal circuits that exist in brain regions vital to anxiety. As the neuroimmune system is becoming a popular target for the treatment of alcohol use disorders (Erickson et al., 2019) and anxiety disorders (Li et al., 2015) it is important to focus on the neurons as well as the glia.

## Data Availability Statement

The raw data supporting the conclusions of this article will be made available by the authors, without undue reservation.

## Ethics Statement

The animal study was reviewed and approved by UNC Chapel Hill Institutional Animal Use and Care Committee.

## Author Contributions

KH, DK, LA, HC, and GB designed the experiments. KH, DK, CT, and HC conducted the experiments. IB and LA contributed the virus. KH, CT, and HC analyzed the data and prepared the figures. KH wrote the manuscript. IB wrote the Supplementary Methods. DK, LA, HC, IB, and GB edited the manuscript. All authors contributed to the article and approved the submitted version.

## Conflict of Interest

The authors declare that the research was conducted in the absence of any commercial or financial relationships that could be construed as a potential conflict of interest.

## References

[B2] Adler M. W. Geller E. B. Chen X. Rogers T. J. (2006). Viewing chemokines as a third major system of communication in the brain. AAPS. J. 7, E865–E870. 10.1208/aapsj070484 16594639 PMC2750956

[B1] Aurelian L. Balan I. (2019). GABA_A_ R α2-activated neuroimmune signal controls binge drinking and impulsivity through regulation of the CCL2/CX3CL1 balance. Psychopharmacology 236, 3023–3043. 10.1007/s00213-019-05220-4 31030249

[B4] Banisadr G. Gosselin R.-D. Mechighel P. Kitabgi P. Rostene W. Parsadaniantz S. M. (2005a). Highly regionalized neuronal expression of monocyte chemoattractant protein-1 (MCP-1/CCL2) in rat brain: evidence for its colocalization with neurotransmitters and neuropeptides. J. Comp. Neurol. 489, 275–292. 10.1002/cne.20598 16025454

[B5] Banisadr G. Gosselin R.-D. Mechighel P. Rostene W. Kitabgi P. Parsadaniantz S. M. (2005b). Constitutive neuronal expression of CCR2 chemokine receptor and its colocalization with neurotransmitters in normal rat brain: functional effect of MCP-1/CCL2 on calcium mobilization in primary cultured neurons. J. Comp. Neurol. 492, 178–192. 10.1002/cne.20729 16196033

[B6] Baxter-Potter L. N. Henricks A. M. Berger A. L. Bieniasz K. V. Lugo J. M. McLaughlin R. J. (2017). Alcohol vapor exposure differentially impacts mesocorticolimbic cytokine expression in a sex-, region- and duration-specific manner. Neuroscience. 346, 238–246. 10.1016/j.neuroscience.2017.01.015 28131626

[B7] Bertotto M. E. Bussolino D. F. Molina V. A. Martijena I. D. (2010). Increased voluntary ethanol consumption and c-Fos expression in selected brain areas induced by fear memory retrieval in ethanol withdrawn rats. Eur. Neuropsychopharmacol. 20, 568–581. 10.1016/j.euroneuro.2010.02.014 20400272

[B10] Blednov Y. A. Bergeson S. E. Walker D. Ferreira V. M. M. Kuziel W. A. Harris R. A. (2005). Perturbation of chemokine networks by gene deletion alters the reinforcing actions of ethanol. Behav. Brain Res. 165, 110–125. 10.1016/j.bbr.2005.06.026 16105698 PMC3040067

[B8] Bonassoli V. T. Contardi E. B. Milani H. de Oliveira R. M. W. (2013). Effects of nitric oxide synthase inhibition in the dorsolateral periaqueductal gray matter on ethanol withdrawal-induced anxiety-like behavior in rats. Psychopharmacology 228, 487–498. 10.1007/s00213-013-3049-1 23494233

[B9] Bonassoli V. T. Milani H. de Oliveira R. M. W. (2011). Ethanol withdrawal activates nitric oxide-producing neurons in anxiety-related brain areas. Alcohol 45, 641–652. 10.1016/j.alcohol.2010.11.007 21194876

[B3] Breder C. D. Tsujimoto M. Terano Y. Scott D. W. Saper C. B. (1993). Distribution and characterization of tumor necrosis factor-alpha-like immunoreactivity in the murine central nervous system. J. Comp. Neurol. 337, 543–567. 10.1002/cne.903370403 8288770

[B11] Ehrlich D. Pirchl M. Humpel C. (2012). Effects of long-term moderate ethanol and cholesterol on cognition, cholinergic neurons, inflammation and vascular impairment in rats. Neuroscience. 205, 154–166. 10.1016/j.neuroscience.2011.12.054 22244974 PMC3314917

[B13] Eisenhardt M. Hansson A. C. Spanagel R. Bilbao A. (2015). Chronic intermittent ethanol exposure in mice leads to an up-regulation of CRH/CRHR1 signaling. Alcohol Clin. Exp. Res. 39, 752–762. 10.1111/acer.12686 25833034

[B12] Erickson E. K. Grantham E. K. Warden A. S. Harris R. A. (2019). Neuroimmune signaling in alcohol use disorder. Pharmacol. Biochem. Behav. 177, 34–60. 10.1016/j.pbb.2018.12.007 30590091 PMC6946054

[B14] File S. E. Seth P. (2003). A review of 25 years of the social interaction test. Eur. J. Pharmacol. 463, 35–53. 10.1016/s0014-2999(03)01273-1 12600701

[B16] Freeman K. Brureau A. Vadigepalli R. Staehle M. M. Brureau M. M.. (2012). Temporal changes in innate immune signals in a rat model of alcohol withdrawal in emotional and cardiorespiratory homeostatic nuclei. J. Neuroinflammation. 9:97. 10.1186/1742-2094-9-97 22626265 PMC3411448

[B15] Frye G. D. McCown T. J. Breese G. R. (1983). Characterization of susceptibility to audiogenic seizures in ethanol-dependent rats after microinjection of γ-aminobutyric acid (GABA) agonists into the inferior colliculus, substantia nigra or medial septum. J. Pharmacol. Exp. Ther. 227, 663–670. 6317842 PMC3310216

[B18] García-Marchena N. Araos P. F. Barrios V. Sánchez-Marín L. Chowen J. A. Pedraz M.. (2017). Plasma chemokines in patients with alcohol use disorders: association of CCL11 (Eotaxin-1) with psychiatric comorbidity. Front. Psychiatry. 7:214. 10.3389/fpsyt.2016.00214 28149283 PMC5242327

[B17] Gruol D. L. (2016). Impact of increased astrocyte expression of IL-6, CCL2 or CXCL10 in transgenic mice on hippocampal synaptic function. Brain. Sci. 6. 10.3390/brainsci6020019 27322336 PMC4931496

[B19] Gungor N. Z. Paré D. (2016). Functional heterogeneity in the bed nucleus of the stria terminalis. J. Neurosci. 36, 8038–8049. 10.1523/JNEUROSCI.0856-16.2016 27488624 PMC4971356

[B21] Harper K. M. Knapp D. J. Breese G. R. (2015). Withdrawal from chronic alcohol induces a unique CCL2 mRNA increase in adolescent but not adult brain—relationship to blood alcohol levels and seizures. Alcohol Clin. Exp. Res. 39, 2375–2385. 10.1111/acer.12898 26556523 PMC4712108

[B22] Harper K. M. Knapp D. J. Park M. A. Breese G. R. (2017). Age-related differences in anxiety-like behavior and amygdalar CCL2 responsiveness to stress following alcohol withdrawal in male wistar rats. Psychopharmacology 234, 79–88. 10.1007/s00213-016-4439-y 27665607 PMC5203962

[B23] Harper K. M. Knapp D. J. Park M. A. Breese G. R. (2018). Differential effects of single versus repeated minocycline administration-lack of significant interaction with chronic alcohol history. Pharmacol. Biochem. Behav. 168, 33–42. 10.1016/j.pbb.2018.03.007 29572015

[B20] He J. Crews F. T. (2008). Increased MCP-1 and microglia in various regions of the human alcoholic brain. Exp. Neurol. 210, 349–358. 10.1016/j.expneurol.2007.11.017 18190912 PMC2346541

[B24] Huang M. M. Overstreet D. H. Knapp D. J. Angel R. Wills T. A. Navarro M.. (2010). Corticotropin-releasing factor (CRF) sensitization of ethanol withdrawal-induced anxiety-like behavior is brain site specific and mediated by CRF-1 receptors: relation to stress-induced sensitization. J. Pharmacol. Exp. Ther. 332, 298–307. 10.1124/jpet.109.159186 19843974 PMC2802475

[B25] June H. L. Liu J. Warnock K. T. Bell K. A. Balan I. Bollino D.. (2015). CRF-amplified neuronal TLR4/MCP-1 signaling regulates alcohol self-administration. Neuropsychopharmacology. 40, 1549–1559. 10.1038/npp.2015.4 25567426 PMC4397415

[B26] Kane C. J. M. Phelan K. D. Douglas J. C. Wagoner G. Johnson J. W. Xu J.. (2014). Effects of ethanol on immune response in the brain: region-specific changes in adolescent versus adult mice. Alcohol. Clin. Exp. Res. 38, 384–391. 10.1111/acer.12244 24033454 PMC3872252

[B29] Kapcala L. P. Dicke J. A. (1992). Brain corticotropin-releasing hormone receptors on neurons and astrocytes. Brain Res. 589, 143–148. 10.1016/0006-8993(92)91174-d 1330205

[B27] Knapp D. J. Whitman B. A. Wills T. A. Angel R. A. Overstreet D. H. Criswell H. E.. (2011). Cytokine involvement in stress may depend on corticotrophin releasing factor to sensitize ethanol withdrawal anxiety. Brain. Behav. Immun. 1, S146–S154. 10.1016/j.bbi.2011.02.018 21377524 PMC3138123

[B28] Knapp D. J. Harper K. M. Whitman B. A. Zimomra Z. Breese G. R. (2016). Stress and withdrawal from chronic ethanol induce selective changes in neuroimmune mRNAS in differing brain sites. Brain. Sci 6. 10.3390/brainsci6030025 27472367 PMC5039454

[B30] Li B. Gao T. Du J. (2015). Neuroimmune imbalance: the key for the treatment of anxiety? J. Immunol. Sci. 16, 317–331. 10.29245/2578-3009/2019/3.117526655061

[B31] Lim J. C. Lu W. Beckel J. M. Mitchell C. H. (2016). Neuronal release of cytokine IL-3 triggered by mechanosensitive autostimulation of the P2X7 receptor is neuroprotective. Front. Cell. Neurosci. 10:270. 10.3389/fncel.2016.00270 27932954 PMC5120082

[B32] Overstreet D. H. Knapp D. J. Breese G. R. (2002). Accentuated decrease in social interaction in rats subjected to repeated ethanol withdrawals. Alcohol Clin. Exp. Res. 26, 1259–1268. 10.1097/01.ALC.0000023983.10615.D7 12198403 PMC2865239

[B340] Reynolds E. S. (1963). The use of lead citrate at high pH as an electron-opaque stain in electron microscopy. J. Cell Biol. 17, 208–212. 10.1083/jcb.17.1.208 13986422 PMC2106263

[B33] Sawada A. Niiyama Y. Ataka K. Nagaishi K. Yamakage M. Fujimiya M. (2014). Suppression of bone marrow-derived microglia in the amygdala improves anxiety-like behavior induced by chronic partial sciatic nerve ligation in mice. Pain. 155, 1762–1772. 10.1016/j.pain.2014.05.031 24907405

[B34] Shackman A. J. Fox A. S. (2016). Contributions of the central extended amygdala to fear and anxiety. J. Neurosci. 36, 8050–8063. 10.1523/JNEUROSCI.0982-16.2016 27488625 PMC4971357

[B35] Stevens S. L. Shaw T. E. Dykhuizen E. Lessov N. S. Hill J. K.. (2003). Reduced cerebral injury in CRH-R1 deficient mice after focal ischemia: a potential link to microglia and atrocytes that express CRH-R1. J. Cereb. Blood Flow Metab. 23, 1151–1159. 10.1097/01.wcb.0000086957.72078.d4 14526225

[B36] Tarozzo G. Bortolazzi S. Crochemore C. Chen S.-C. Lira A. S. Abrams J. S.. (2003). Fractalkine protein localization and gene expression in mouse brain. J. Neurosci. Res. 73, 81–88. 10.1002/jnr.10645 12815711

[B38] Valenta J. P. Gonzales R. A. (2016). Chronic intracerebroventricular infusion of monocyte chemoattractant protein-1 leads to a persistent increase in sweetened ethanol consumption during operant self-administration but does not influence sucrose consumption in long-evans rats. Alcohol Clin. Exp. Res. 40, 187–195. 10.1111/acer.12928 26683974 PMC4701601

[B39] van Gassen K. L. I. Netzeband J. G. de Graan P. N. E. Gruol D. L. (2005). The chemokine CCL2 modulates Ca^2+^ dynamics and electrophysiological properties of cultured cerebellar Purkinje neurons. Eur. J. Neurosci. 21, 2949–2957. 10.1111/j.1460-9568.2005.04113.x 15978006

[B37] Van Steenwinckel J. Goazigo R.-L. Pommier P. Mauborgne A. Dansereau M.-A. Kitabgi P.. (2011). CCL2 released from neuronal synaptic vesicles in the spinal cord is a major mediator of local inflammation and pain after peripheral nerve injury. J. Neurosci. 31, 5865–5875. 10.1523/jneurosci.5986-10.2011 21490228 PMC6622829

[B40] Vetreno R. P. Qin L. Crews F. T. (2013). Increased receptor for advanced glycation end product expression in the human alcoholic prefrontal cortex is linked to adolescent drinking. Neurobiol. Dis. 59, 52–62. 10.1016/j.nbd.2013.07.002 23867237 PMC3775891

[B43] Wakida N. Kiguchi N. Saika F. Nishiue H. Kobayashi Y. Kishioka S. (2014). CC-chemokine ligand 2 facilitates conditioned place preference to methamphetamine through the activation of dopamine systems. J. Pharmacol. Sci. 125, 68–73. 10.1254/jphs.14032fp 24748435

[B41] Whitman B. A. Knapp D. J. Werner D. F. Crews F. T. Breese G. R. (2013). The cytokine mRNA increase induced by withdrawal from chronic ethanol in the sterile environment of brain is mediated by CRF and HMGB1 release. Alcohol Clin. Exp. Res. 37, 2086–2097. 10.1111/acer.12189 23895427 PMC3815509

[B44] Wills T. A. Knapp D. J. Overstreet D. H. Breese G. R. (2008). Differential dietary ethanol intake and blood ethanol levels in adolescent and adult rats: effects on anxiety-like behavior and seizure thresholds. Alcohol Clin. Exp. Res. 32, 1350–1360. 10.1111/j.1530-0277.2008.00709.x 18540921 PMC2855489

[B45] Wills T. A. Knapp D. J. Overstreet D. H. Breese G. R. (2010). Interactions of stress and CRF in ethanol-withdrawal induced anxiety in adolescent and adult rats. Alcohol Clin. Exp. Res. 34, 1603–1612. 10.1111/j.1530-0277.2010.01245.x 20586753 PMC2948468

[B42] Wohleb E. S. Powell N. D. Godbout J. P. Sheridan J. F. (2013). Stress-induced recruitment of bone marrow-derived monocytes to the brain promotes anxiety-like behavior. J. Neurosci. 33, 13820–13833. 10.1523/jneurosci.1671-13.2013 23966702 PMC3755721

[B46] Zhou Y. Tang H. Liu J. Dong J. Xiong H. (2011). Chemokine CCL2 modulation of neuronal excitability and synaptic transmission in rat hippocampal slices. J. Neurochem. 116, 406–414. 10.1111/j.1471-4159.2010.07121.x 21105875 PMC3018532

