# Supplementary material for: Phenotyping CCL2 Containing Central Amygdala Neurons Controlling Alcohol Withdrawal-Induced Anxiety
Source: Front Cell Neurosci. 2020 Sep 18;14:580583. doi: 10.3389/fncel.2020.580583 (PMC7531233; doi:10.3389/fncel.2020.580583)
Supplement: Supplementary file 1 [file Table_1.DOCX]

**Phenotyping CCL2 containing central amygdala neurons controlling alcohol withdrawal induced anxiety**

Kathryn M Harper, PhD, Darin J Knapp, PhD, Caroline A Todd, Irina Balan, PhD, Laure Aurelian, PhD, Hugh E Criswell, PhD, and George R Breese, PhD

**Supplemental Materials & Methods**

*Small Interfering RNAs:* Small interfering (si) RNA was designed to target the nucleotide (nt) -6-13 sequence within the rat CCL2 gene (Gene bank Entry No. NM_031530). A scrambled siRNA (siNC) served as control. BLAST search against EST libraries was performed to ensure that no other gene was targeted. The CCL2 siRNA is ACCACTATGCAGGTCTCTG^1^, and the scrambled siRNA is TAACGACGCGACGACGTAA. The siRNA was synthesized as 60-mer sense and antisense oligonucleotide templates (19 × 2 nt) specific to the targeted gene and 22 nt for restriction enzyme sites and hairpin structure. Synthesis was at the University of Maryland Biopolymer Core Facility and used the phosphoramidite (AB) technology. Inhibition of cognate gene expression was confirmed in RAW264.7 cells that express CCL2, as previously described^2^. Specifically, the siRNA was transfected at a final concentration of 65 nM using the siPORT amine transfection agent (Thermo Fisher Scientific, Cat. # AM4502) according to the manufacturer’s instructions, and extracts collected 72 h post-transfection were assayed for protein content by the BCA procedure (Thermo Fisher Scientific). The in vitro 3,3′,5,5′-tetramethylbenzidine (TMB) based enzyme-linked immunosorbent assay (ELISA) for the measurement of CCL2 was done using the CCL2/MCP-1 ELISA kit (Cat. #ERC-MCP-1-CL; Raybiotech, Norcross, GA, USA), as per manufacturer’s instructions.

*HSV-1–Based Amplicon Vectors:* siRNAs were delivered with non-replicating non-toxic herpes simplex virus type 1 (HSV-1) vectors, known as amplicons. Amplicons are bacterial plasmids that contain two noncoding elements from HSV-1, an origin of DNA replication and a DNA packaging/cleavage signal, which allow replication and packaging into HSV-1 particles as a 150-kb concatamer. Numerous copies of the transgene sequences are packaged into one vector particle, thereby allowing for high expression levels. Amplicons retain the HSV naturally discriminative in vivo neurotropism and their use in specific siRNA-mediated gene knockdown was previously described^1-5^. Briefly, the pHSVsi vector used to generate the siRNA plasmids that are packaged into HSV-1 virions expresses EGFP under the direction of the HSV-1 IE4/5 immediate-early promoter. The incorporation of EGFP allows for the titration of the vector stocks and the visualization of cell transduction in culture and in the CNS. The pSUPER plasmid, which contains the RNA polymerase III-dependent H1 promoter and well-defined start of transcription and termination signals, is used to generate a second transcription unit for the synthesis of siRNA. The siRNAs were inserted into the pHSVsi vector between the BglII and HindIII sites, downstream of the RNA polymerase III-dependent H1 promoter and packaged as previously described^1-5^. The amplicon titers were 2.9 x 10^8^ and 2 x 10^8^ Transducing Units (TU)/ml for the amplicons for CCL2 siRNA (pHSVsiCCL2) and scrambled siRNA (pHSVsiNC), respectively.

1 June, H. L. *et al.* CRF-amplified neuronal TLR4/MCP-1 signaling regulates alcohol self-administration. *Neuropsychopharmacology* **40**, 1549-1559, doi:10.1038/npp.2015.4 (2015).

2 Liu, J. *et al.* Binge alcohol drinking is associated with GABAA alpha2-regulated Toll-like receptor 4 (TLR4) expression in the central amygdala. *Proc Natl Acad Sci U S A* **108**, 4465-4470, doi:10.1073/pnas.1019020108 (2011).

3 Aurelian, L., Warnock, K. T., Balan, I., Puche, A. & June, H. TLR4 signaling in VTA dopaminergic neurons regulates impulsivity through tyrosine hydroxylase modulation. *Translational psychiatry* **6**, e815, doi:10.1038/tp.2016.72 (2016).

4 Balan, I., Warnock, K. T., Puche, A., Gondre-Lewis, M. C. & Aurelian, L. Innately activated TLR4 signal in the nucleus accumbens is sustained by CRF amplification loop and regulates impulsivity. *Brain Behav Immun* **69**, 139-153, doi:10.1016/j.bbi.2017.11.008 (2018).

5 Saydam, O. *et al.* Herpes simplex virus 1 amplicon vector-mediated siRNA targeting epidermal growth factor receptor inhibits growth of human glioma cells in vivo. *Molecular therapy : the journal of the American Society of Gene Therapy* **12**, 803-812, doi:10.1016/j.ymthe.2005.07.534 (2005).
