# Supplementary material for: Phenotyping CCL2 Containing Central Amygdala Neurons Controlling Alcohol Withdrawal-Induced Anxiety
Source: Front Cell Neurosci. 2020 Sep 18;14:580583. doi: 10.3389/fncel.2020.580583 (PMC7531233; doi:10.3389/fncel.2020.580583)
Supplement: Supplementary file 2 [file Table_2.DOCX]

Supplemental Table 1: The amount of alcohol consumed was not altered by vector type injected into the CeA.

| Group | First block  (Ave g/kg/day) | Second block  (Ave g/kg/day) | Third block  (Ave g/kg/day) |
| --- | --- | --- | --- |
| ED-Sc | 10.76±.3 | 11.01±.35 | 9.54±.37 |
| ED-CCL2 | 10.8±.27 | 11.11±.49 | 9.71±.46 |

Data are presented as mean±SEM

Supplemental Figure 1: There was no difference in weight gain across groups. Data are presented as mean±SEM
